# HealthCall for the smartphone: technology enhancement of brief intervention in HIV alcohol dependent patients

**DOI:** 10.1186/1940-0640-9-5

**Published:** 2014-02-17

**Authors:** Deborah S Hasin, Efrat Aharonovich, Eliana Greenstein

**Affiliations:** 1 New York State Psychiatric Institute, New York, NY 10032, USA; 2 Department of Psychiatry, Columbia University Medical Center, New York, NY 10032, USA; 3 Department of Epidemiology, Mailman School of Public Health, Columbia University, New York, NY 10032, USA

**Keywords:** HIV, Alcohol, Brief intervention, Technology intervention, Smartphone, Primary care

## Abstract

**Background:**

Heavy drinking jeopardizes the health of patients in HIV primary care. In alcohol dependent patients in HIV primary care, a technological enhancement of brief intervention, HealthCall administered via interactive voice response (HealthCall-IVR) was effective at reducing heavy drinking. The smartphone offered a technology platform to improve HealthCall.

**Methods:**

Working with input from patients, technology experts, and HIV clinic personnel, we further developed HealthCall, harnessing smartphone technological capacities (HealthCall-S). In a pilot study, we compared rates of HealthCall-S daily use and drinking outcomes in 41 alcohol dependent HIV-infected patients with the 43 alcohol dependent HIV-infected patients who used HealthCall-IVR in our previous efficacy study. Procedures, clinic, personnel, and measures were largely the same in the two studies, and the two groups of patients were demographically similar (~90% minority).

**Results:**

Pilot patients used HealthCall-S a median of 85.0% of the 60 days of treatment, significantly greater than the corresponding rate (63.8%) among comparison patients using HealthCall-IVR (p < .001). Mean end-of-treatment drinks per drinking day was similar in the two groups. Patients were highly satisfied with HealthCall-S (i.e., 92% reported that they liked using HealthCall-S).

**Conclusions:**

Among alcohol dependent patients in HIV primary care, HealthCall delivered via smartphone is feasible, obtains better patient engagement than HealthCall-IVR, and is associated with decreased drinking. In HIV primary care settings, HealthCall-S may offer a way to improve drinking outcomes after brief intervention by extending patient engagement with little additional demands on staff time.

## Background

Alcohol has been termed “the forgotten drug” in the HIV epidemic [1] and heavy or hazardous drinking is important to target in improving the health and survival of those infected with HIV [2]. Heavy drinking is associated with poor antiretroviral (ART) adherence [3-5], is a leading cause of morbidity and mortality [6-10] among those with liver disease, and is a clinical challenge for those with HIV [11,12]. Despite calls to address this [8,13-16], heavy drinking remains prevalent in HIV clinic settings [13,15], where resources for interventions are often limited [17-19]. Recommendations to refer patients to outside treatment [11,20] do not solve this, since patients seldom follow referrals [21,22]. Feasible, scalable drinking-reduction interventions for use within the HIV clinic are needed.

Trials in HIV populations [23-27] show that some interventions are effective for drinking-reduction [25-27]. However, their length (6–15 sessions, 540–1350 min), limits dissemination potential. In general primary care patients with hazardous, non-dependent drinking, brief interventions are effective (from structured advice to well-supervised motivational interviewing [MI]) [22,28-34]. However, primary care patients with severe drinking problems need more extensive intervention [22,34-36]. The problem is how to extend the intervention “dose” without unrealistically increasing staff time.

**Figure 1:**
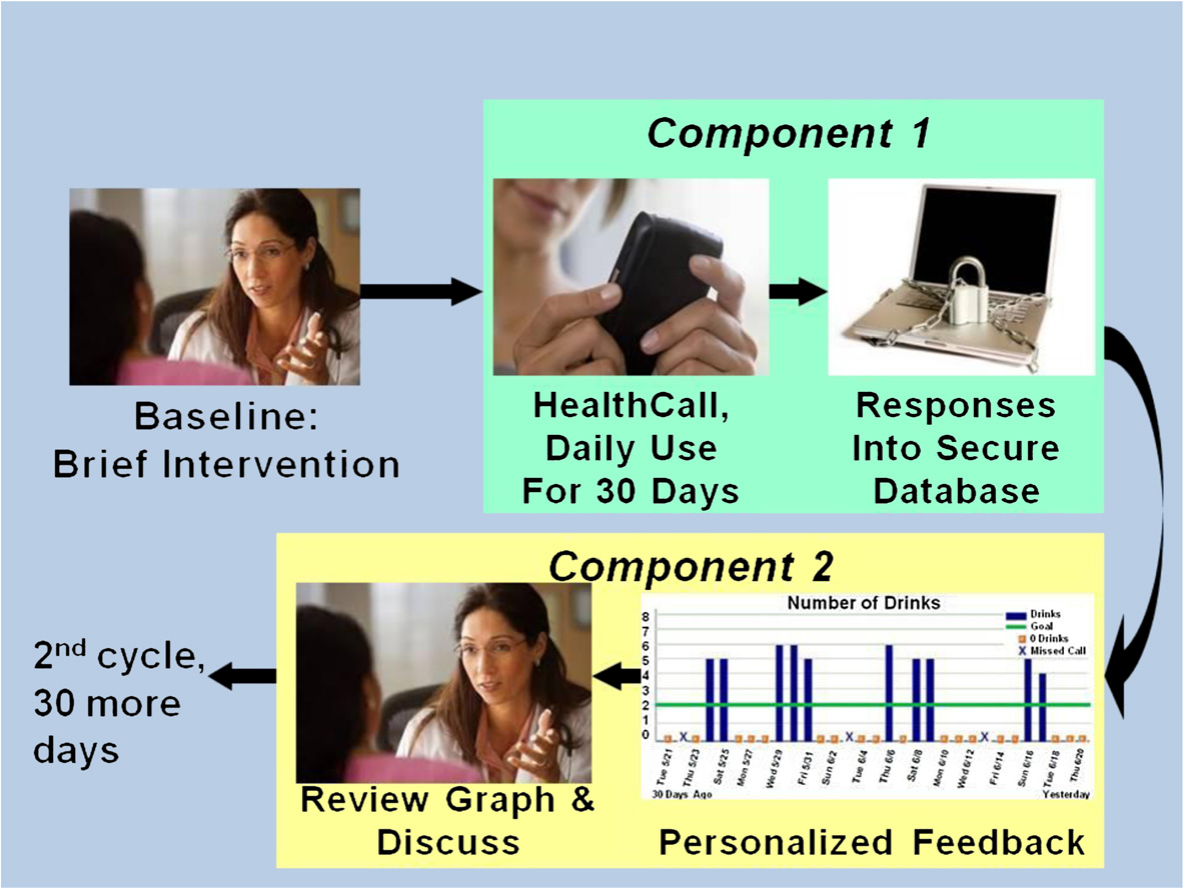
The two components of HealthCall.

User-friendly mobile technologies can extend patient involvement without greatly increasing staff time. Using interactive voice response (IVR) technology, we developed the initial version of “HealthCall” [35-37] to extend HIV patient involvement after a brief drinking-reduction intervention. HealthCall is explained to the patient at the end of the brief session. HealthCall has two main components, each delivering an evidence-based change technique (Figure 1).

(1) Component 1 delivers *self*-*monitoring*. Patients self-monitor drinking for 60 days by answering brief (2–4 min) automated questions about drinking and other general and HIV behaviors, and receive reinforcement for doing so (e.g., “We’re glad you called”). Patients’ answers go into a database. Meta-analysis indicates that self-monitoring is a key contributor to the effectiveness of brief intervention [38].

(2) Component 2 delivers *personalized feedback*, also shown by meta-analysis to be an important element in behavior change [39,40]. At 30 and 60 days, a graph of the patient’s drinking and summary of other self-monitoring data is presented to the patient by the staff member. This forms the basis of a 10–15 minute discussion, identifying patterns and planning for ways to improve. Incorporation of an in-person meeting is consistent with participants’ preference for technology-based interventions that are combined with interpersonal support [41], and with a meta-analysis indicating that interventions involving some personal contact are more effective than interventions that are entirely electronically delivered [42].

To reduce hazardous drinking in patients in urban HIV primary care, we tested HealthCall on the IVR platform (HealthCall-IVR) in a large randomized trial [35], comparing MI + HealthCall-IVR to MI-only and a time-matched educational control. Patients in MI + HealthCall-IVR made a median of 64.4% of the 60 HealthCall daily calls. At the end of treatment, drinking was significantly lower in MI + HealthCall-IVR than MI-only or control, with results entirely concentrated within alcohol dependent patients [35]. The trial showed that HealthCall successfully enhanced brief drinking-reduction intervention among alcohol dependent patients in HIV primary care, while suggesting room for improved engagement in daily self-monitoring.

Smartphone costs are decreasing and use is increasing rapidly [43,44], suggesting that the diffusion of smartphone technology can provide widespread, engaging HIV interventions [45]. For HealthCall, compared to IVR, smartphone technology has three main advantages to engage patients: 1) visuals and graphics: the screen can offer images, including graphics and a video of a friendly counselor delivering greetings, questions, reinforcement, and suggestions. The visual aspects seemed likely to help engage cognitively impaired patients. (Cognitive deficits are associated with alcohol dependence, greater viral load [46-48] and treatment dropout [49,50], so cognitive considerations underlie our HealthCall work); 2) accessibility and connectivity: HealthCall on the smartphone (HealthCall-S) offers immediate access any time, regardless of telephone or internet availability; 3) more nuanced interactivity: due to the greater smartphone computing capacity, HealthCall-S could be designed to provide more complex patterns of questions or statements more closely corresponding to patients’ answers to previous questions.

Given these advantages, we conducted a pilot study to adapt HealthCall for delivery via on the smartphone (HealthCall-S) among alcohol dependent patients in HIV primary care, and determine HealthCall-S feasibility in terms of patient engagement, drinking reduction, and reactions. To maximize the information about HealthCall-S with the resources available, we used Android smartphones and conducted a single-arm study of HealthCall-S. To place the results in context, we compared HealthCall-S results on patient engagement and drinking reduction to results we previously obtained using HealthCall-IVR in HIV alcohol dependent patients at the same clinic, who served as a historical comparison group (hereafter, “comparison group”). The comparison group consisted of the alcohol dependent patients in the MI + HealthCall-IVR arm of our previous randomized trial [35]. Aside from a pilot study requirement that patients meet criteria for alcohol dependence and the fact that we provided each patient with an Android smartphone, the setting, other eligibility criteria, drinking measures, and study procedures were held constant in this pilot study and the randomized trial [35].

## Methods

### HealthCall-S adaptation process

We obtained patient and HIV health educator-counselor input throughout this process to create a more engaging, user-friendly patient procedure. All patients giving input were minority individuals with alcohol or drug abuse treated at the urban HIV primary care clinic where we conducted our studies. We used the Android rather than iPhone platform because Android is less expensive.

### Stage 1

A bilingual MI + HealthCall counselor conducted individual interviews with five patients who previously used HealthCall-IVR to ask general reactions to HealthCall for the smartphone, and to specific ideas for changes. Patients responded favorably, and made suggestions (e.g., more personalization, variation to make calls more interesting, adding a convenient way to request discussion with the counselor).

### Stage 2

Using these and other ideas gained from the original randomized trial, a draft of the HealthCall-S daily script was prepared, using the HealthCall-IVR script as a starting point. Changes included new questions and statements for a video counselor, graphic images to illustrate statements and questions, and greater personalization to patients’ responses.

### Stage 3

In a focus group, five previous HealthCall-IVR participants gave their reactions to specific aspects of the script. Topics included preference for real or animated video counselor (video preferred by all), interest in a graph of drinking over the prior seven days (4 of 5 patients wanted this, and suggested making it optional), preferences for different images, and question wording. This information was used to refine the script.

### Stage 4

The research team worked with Andriod programmers on how to use smartphone capacities to implement the script in the most user-friendly, engaging way. The script was further developed collaboratively with the programming experts and HIV health educators who worked on HealthCall studies.

### Stage 5

Video portions of the script were produced (versions in English and Spanish), with a bi-lingual counselor as the actress. These were edited via collaboration between the research team, programming experts, and the medical center media department.

### Stage 6

Consulting closely with the research team, the programmers prepared a beta version of HealthCall-S, integrating the images, video, and an oversized keypad for easy entry of patient responses. The research team then extensively pre-tested of the beta version, which was debugged by the programmers.

### Stage 7

A counselor conducted individual interviews with six previous participants in HealthCall studies to obtain detailed reactions to each component of HealthCall-S. Based on these reactions, the research staff made final adjustments to the HealthCall-S script, which were implemented by the programmers.

### Pilot study procedures

**Figure 2:**
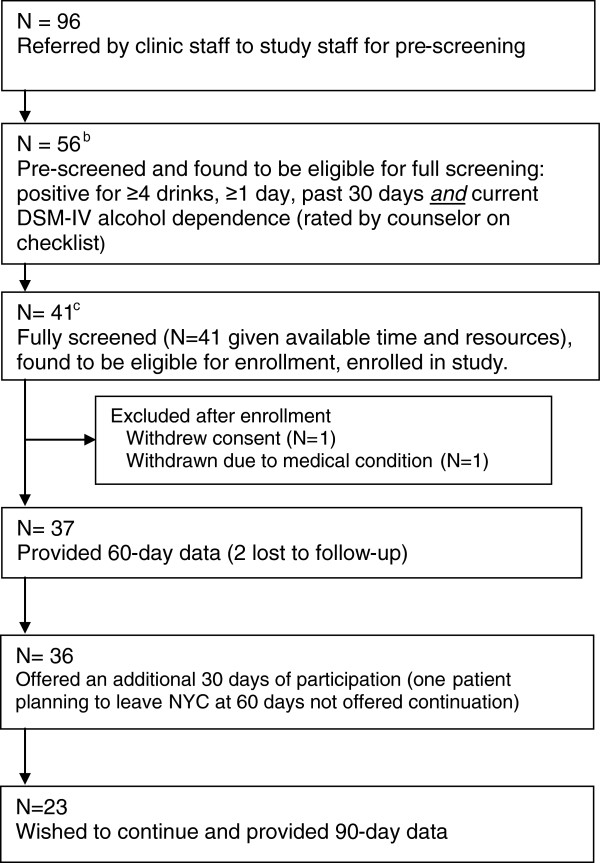
**Flowchart of pilot study participants **^**a**^**. **^a^Study of HealthCall-Smartphone (HealthCall-S) enhancement of Motivational Interviewing for drinking reduction: New York City HIV primary care alcohol dependent patients at baseline; patients enrolled June 2012-July 2012. ^b^Drinking eligibility (≥4 drinks, ≥1 day, past 30 days) identical to the previous 3-arm MI + Healthcall-IVR randomized trial [35]; alcohol dependence an additional eligibility requirement. ^c^ Other eligibility requirements included absence of psychosis, being actively suicidal or homicidal, severely cognitive impaired using Halstead-Reitan Trails A. These requirements were identical to the previous 3-arm MI + Healthcall-IVR randomized trial [35].

Pilot study patients were recruited from a large urban HIV primary care clinic. Eligibility included ≥4 drinks of alcohol at least once in the prior 30 days; current DSM-IV alcohol dependence; English- or Spanish-speaking; ≥18 years; not actively psychotic, suicidal, homicidal, or grossly cognitively impaired; and not a participant in the prior randomized trial [35]. Clinic staff routinely asked all patients an electronic chart question about current drinking, and referred patients likely to meet the drinking threshold to the bilingual counselors for eligibility assessment and screening, informed consent, assessment, and intervention in English or Spanish. Between June 2012 and July 2012, counselors pre-screened 96 patients for eligibility, using a paper-and-pencil checklist that covered (a) occasions of ≥4 drinks of alcohol in the prior 30 days and (b) current DSM-IV alcohol dependence criteria. Of these, 56 patients passed the pre-screen procedures. Of the 56, 41 patients were fully screened, including counselor administration of the Halstead-Reitan Trials A test [51], and counselor computer-assisted psychosis items from the Structured Clinical Interview for DSM-IV (SCID; [52]), suicide and homicide items from the Addiction Severity Index [53], and DSM-IV alcohol dependence items from the NIAAA Alcohol Use Disorders and Associated Disabilities Interview Schedule (AUDADIS-IV), a reliable and valid diagnostic procedure in the general population and in English- and Spanish-speaking substance abuse and primary care patients [54-56]. Counselors also cross-checked patients’ clinic charts for evidence of psychosis, suicidality, or homicidality. All 41 of the fully screened patients were found to be eligible, completed a full assessment battery (see below) and entered into the pilot study (Figure 2). Due to organizational constraints (ending of a clinic sub-contract), we did not conduct formal eligibility screening of the remaining 15 patients.

### Comparison group procedures

Comparison group patients were recruited earlier for the randomized trial [35] from the same clinic, by the same coordinator, with the same procedures and eligibility criteria, with one exception: alcohol dependence was not an eligibility requirement in that study, so the alcohol dependence pre-screening procedures were not employed. In that study, the identical A-CASI method was used to assess DSM-IV alcohol dependence criteria among all patients entered. Patients in the MI + HealthCall-IVR arm who met criteria for DSM-IV alcohol dependence constituted the comparison group (N = 43).

Patients in both studies were assessed at baseline, received a 20–25 min MI, were instructed in HealthCall use, told that it might help them reduce their drinking, asked to use HealthCall for 60 days, and asked to return for assessment and brief counselor meetings at 30 and 60 days. To explore HealthCall-S over a longer period, pilot study patients were offered 30 more days of participation at 60 days. Columbia University and Mt. Sinai Medical Center IRBs approved all procedures.

### Assessments

During treatment, measures were administered (in English or Spanish) prior to the brief counselor meetings, using audio computer-assisted self-interviews (A-CASI). Alcohol consumption was assessed with A-CASI 30-day TimeLine FollowBacks (TLFB) [57].

We measured patient perceptions and satisfaction with HealthCall-S participation with a set of 12 items we designed to tap reactions to specific aspects of HealthCall-S. Three- or five-level Likert-type items covered such aspects as patients’ feelings of safety and privacy using HealthCall-S, whether it affected their memory and understanding of their drinking patterns, motivation and self-confidence in drinking reduction efforts, and whether it reminded them of their drinking goal and initial counselor meeting. Patients self-administered these questions after their final meeting with their counselor. Responses were grouped into three levels across all items for ease of interpretation.

### Outcomes

The primary drinking outcome for both studies was mean number of drinks per drinking day in the prior 30 days (NumDD), created with TLFB data. NumDD was selected due to the potential for liver toxicity and damage from large alcohol quantities. A secondary outcome was percent days abstinent (PDA) over the prior 30 days.

### Motivational interview

At baseline, counselors administered a 20–25 minute individual MI session using standard techniques to motivate drinking reduction. In the pilot study, three counselors administered the interventions. All had prior experience as HIV health educators, but not as alcoholism counselors. The first two were the MI + HealthCall counselors in the three-arm randomized trial; the third administered MI + HealthCall in a similar study on non-injection drug use [37]. Counselors also gave all patients a NIAAA drinking reduction pamphlet [58].

### HealthCall-S Component 1: Self-Monitoring

An abbreviated version of the HealthCall-S script is shown in Table 1. As shown, the script included a welcoming greeting, questions about quantities of beer, wine and liquor, desire (craving) to drink, whether the patient thought about pros and cons of drinking or his/her drinking goal, the importance of drinking reduction and commitment to this, reasons for drinking or not drinking, a statement praising/reinforcing drinking reduction or continued calling, drug use, use of antiretroviral medication, safe sex, how the patient felt mentally and physically, an offer to see a graph showing the last seven days of drinking, an offer to speak with the counselor, a tip to reduce drinking that varied daily, and a goodbye that had 15 variations.

**Table 1:** **HealthCall**-**Smartphone script for daily self**-**monitoring calls**

**Content of statement or question**	**Purpose**
1. Friendly welcome greeting that varies by day^a^	Reinforce HealthCall-S use; add variety to daily script
2. Request to enter password	Ensure privacy
3. Statement that questions are about yesterday (morning, afternoon, evening or during the night)	Standardize timeframe regardless of when HealthCall-S is used
4. Number of drinks of beer, wine, liquor^a,b^?	*Self*-*monitor drinking to increase self*-*awareness*; provide data for 30- and 60-day personalized feedback
5. How much wanted to drink?	*Self*-*monitor craving to increase self*-*awareness*
6. Optional view of graph showing number of drinks daily over prior 7 days^a,b^	*Self*-*monitor progress to increase self*-*awareness* (optional because not all patients wish to see the graph every day)
7. Thought about pros and cons of drinking^b^? (yes/no)	Remind patient of MI session to *maintain or increase motivation to change*
8. Thought about drinking reduction goal^b^? (yes/no)	Remind patient of MI goal to *maintain or increase motivation to change*
9. How important was drinking reduction?	
10. How committed to drinking reduction?	
11. IF DRANK: Reasons for drinking^a^ (yes/no to nine reasons, e.g., around others who drank; to improve mood; habit)?	Identify drinking motives and triggers (internal and social-contextual) *to increase self*-*awareness*, provide data for 30- and 60-day personalized feedback
12. IF DID NOT DRINK: Reasons for not drinking^a^ (yes/no to nine reasons, e.g., my health; made commitment not to; no money for alcohol; feel good when do something positive for self)?	*Maintain or increase self*-*efficacy and motivation to change*
13. Statement praising abstinence or meeting drinking goal (4 rotated, different statements), *or* continuing to call even if goals weren’t met^a^	*Increase motivation and self*-*efficacy*; reinforce continued HealthCall-S use; add variety to daily script
14. Statement of one of 30 daily “tips” in random order (suggestions on how to cut down drinking or maintain abstinence)^a^	Suggest skills to patients for cutting down or staying abstinent based NIAAA evidence-based materials; also to add variety to daily script
15. Drug use?	Identify potential substitute substance use pattern
16. If on ART, were all HIV meds taken?	Self-monitoring of ART; identify if alcohol and ART adherence are linked
17. If had sex, was it protected^b^?	Self-monitoring of sexual risk behaviors
18. Felt well physically? Stressed? Angry? sad/depressed? how was day overall? (5 yes/no questions)^b^	*Increase self*-*awareness*; identify drinking triggers and patterns; show concern for the whole person
19. If reported at-risk drinking, counselor call offered via one-touch link to counselor’s phone/voicemail^a^	Provide counselor assistance if wished
20. Outgoing tailored goodbye (15 versions)	Reinforce HealthCall-S use based on patients’ input and date/day of the week/weekend; add variety.

^a^question or statement via video of the counselor (others heard and accompanied by text on screen).

^b^accompanied by visual image (photo or drawing) on the screen.

After the MI session, counselors provided patients with an Android smartphone, explained the purpose and use of HealthCall-S and had patients practice using HealthCall-S on the smartphone for the first time to ensure its correct use. A good time for daily use was identified with the patient, and the smartphone alarm set to this time. To start HealthCall-S, patients touched an icon on the smartphone home screen and entered a self-selected password. They input responses on a screen touchpad with numbers enlarged for easier use.

HealthCall-S stored patient input on the smartphone, enabling HealthCall-S use regardless of network connectivity. Responses were transmitted securely to an online server. The database was checked daily for transmitted data. If no data were received for two consecutive days, counselors contacted patients to remind them to continue using HealthCall.

### HealthCall-S Component 2: Personalized feedback

Daily drinking self-monitoring data were used to produce personalized feedback in the form of a graph showing the number of drinks reported daily against the patient’s drinking goal, and summary statistics (average drinks per drinking day; reasons for drinking). After 30 days, counselors met with patients, presented their 1–30 day graph and summary, ensured that they understood it, and used it as the basis for a 10–15 minute discussion of the patient’s drinking. This included identifying patterns, and planning ways to maintain gains or improve. Counselors reinforced drinking reduction and change efforts. Counselors then re-set the drinking goal if patients wished, suggested HealthCall-S use for 30 more days, and scheduled a 60-day meeting. At 60 days, a similar discussion focused on the 31–60 day graph and summary. Pilot patients were offered HealthCall-S use for 30 more days. For those not wishing to continue, the counselor conducted brief termination planning (e.g., encouraging continued self-monitoring). For patients who continued, these procedures were followed 30 more days.

### HealthCall-IVR: comparison group

As described previously [35], self-monitoring was delivered via similar questions on a telephone automated IVR system. Personalized feedback was delivered in an earlier version of the graph and summary, in a similar meeting with the counselor. HealthCall-IVR participation was 60 days, with assessment-only visits at 90 days and subsequently.

### Training, quality control, supervision

A MI Network Trainer (MINT) trained the counselors on MI, giving a refresher course before the pilot study, and made fidelity ratings [59] of six pilot study MI sessions, two for each counselor. These showed good to excellent fidelity across domains, e.g., mean % complex reflections (57.4%), MI spirit/empathy (4.3; 4.0), and number of MI non-adherent statements (0.0). Similar MI fidelity was reported in the three-arm trial [35]. HealthCall procedures were manualized. In both studies, E.A. supervised the counselors weekly on clinical and administrative procedures. No counselor effects on treatment outcome have been detected in any HealthCall studies [35-37].

### Compensation

In the pilot study, patients received a $25 gift certificate at baseline, 30 days, and (if they continued) 90 days. At 60 days, those who terminated received a $40 gift certificate and the smartphone or a $100 gift certificate; continuing patients received a $40 gift certificate. The option to keep the phone or receive a $100 gift certificate was also given at 90 days; its purpose was to discourage sale or loss of the phone during the study. Compensation in the three-arm trial was $20 in gift certificates at each visit up to 90 days. To improve dissemination potential, compensation was not linked to level of HealthCall use.

### Analysis

To compare the two treatment conditions on demographic characteristics, call rates, and dropout rates, chi-square tests were used for categorical variables and Wilcoxon non-parametric tests for non-normally distributed continuous and count variables. Additional generalized linear models that controlled for demographic variables, number of DSM-IV dependence criteria, and number of years since HIV diagnosis were used to additionally test for treatment differences in call rates. To compare NumDD at 60 days in the HealthCall-S and HealthCall-IVR groups, we used a generalized linear model with a negative binomial distribution (PROC GENMOD, SAS), consistent with the method used to examine treatment effects in the three-arm trial. We also explored PDA at 60 days using the same method. All generalized linear models included any participant who provided at least baseline information. The models controlled for baseline number of DSM-IV dependence criteria and number of years since HIV diagnosis, since these differed significantly between the pilot study and comparison groups. Parameter estimates and associated p-values were used to indicate differences between the two conditions. All tests were two tailed: p < 0.05 indicated statistical significance. For the 12 items measuring patient perceptions of and satisfaction with HealthCall-S procedures, we present information descriptively.

## Results

### Participants in the pilot study and comparison group

Of the 41 patients entered into the pilot study (Figure 1), one withdrew (lack of interest) and one was withdrawn (medical condition precluded further participation). Of the 39 remaining patients, 37 remained in the study for at least 60 days and provided 60-day outcome data (25 on time, 12 after the 60 day point). Of these, all but one who planned to leave the area shortly after the 60-day meeting were offered an additional 30 days of intervention; 23 continued for 90 days. Most patients in the pilot study and comparison group were male (71.8% and 81.4%, respectively) and minority (87.1% and 93.1%, respectively); relatively few were employed or in stable relationships (Table 2). Mean baseline drinks per drinking day (NumDD) was high in both groups. Of the characteristics in Table 2, the samples differed in terms of statistical significance on two characteristics: years since HIV diagnosis (more years in the comparison group) and dependence severity (number of DSM-IV criteria, greater in the pilot study group).

**Table 2:** Patient characteristics in pilot study and historical comparison group

	**MI + HealthCall-S**	**MI + HealthCall-IVR**	**p-level**
	**Pilot study**	**Comparison group**	
	**(N = 39)**	**(N = 43)**	
Sociodemographic binary variables^a^	%	%	
Female	28.2	18.6	0.30
Ethnicity			
African American	61.5	51.2	0.34
Hispanic	25.6	41.9	
Other	12.8	07.0	
Spanish-speaking	10.3	11.6	0.84
High school education	74.4	58.1	0.12
Married/Stable relationship	05.1	16.3	0.11
Employed	25.6	11.6	0.10
Residentially instable	30.8	27.9	0.78
Clinical variables			
Drug dependence	10.3	23.3	0.12
Antisocial personality disorder	15.4	20.9	0.52
Beck depression inventory ≥17, indicating clinical depression	08.0	06.7	0.85
Continuous variables^b^	Mean (s.d.)	Mean (s.d.)	
Age, years	45.5 (11.5)	46.0 (7.2)	0.51
Years since HIV diagnosis	11.5 (8.4)	14.9 (7.4)	0.04
Drinks per drinking day (NumDD)	9.3 (6.9)	8.1 (3.9)	0.84
Percent days abstinent (PDA)	58.1 (27.4)	61.3 (24.2)	0.81
DSM-IV dependence criteria count	5.8 (1.1)	4.9 (1.7)	0.03
Beck Depression Inventory	7.5 (6.9)	5.3 (5.9)	0.18

^a^group differences tested with chi-square.

^b^group differences tested with Kruskal Wallis test.

### Exposure to HealthCall-S (pilot study) and HealthCall-IVR (comparison group)

From 1–60 days, pilot patients used HealthCall-S a median of 85.0% of the days, compared to 63.8% of the days in the HealthCall-IVR comparison group, a 33% difference that was statistically significant (Z = 4.76, p < .001). After controlling for demographic variables, preferred language, years since HIV diagnosis, and DSM-IV alcohol dependence criteria count, this difference between pilot and comparison daily use remained statistically significant (X^2^ = 17.66, Numerator_DF_ = 1, Denominator_DF_ = 69, p < 0.001). In the pilot study group, use of HealthCall was similar across patient characteristics, with no significant or clinically meaningful differences found by sex, age, race, education level, marital status, language of participation (English or Spanish), employment status, years since HIV diagnosis, and alcohol severity.

### Retention in treatment: pilot study and comparison group patients

Treatment retention was excellent. In patients using HealthCall-S, 5.1% dropped out by 60 days, while 11.6% of the comparison patients using HealthCall-IVR dropped out by that point (Fisher’s exact test p = 0.44). Although not meeting the threshold for statistical significance, results did favor HealthCall-S.

### Predictors of stopping at 60 days of treatment (pilot study patients)

We examined whether any characteristics in Table 1 predicted stopping at 60 days; no statistically significant or otherwise clinically meaningful relationships were found.

### Change in drinking: HealthCall-S (pilot study) and HealthCall-IVR (comparison group)

Among the pilot study patients, baseline mean NumDD was 9.3, dropping to 3.9 at 60 days, similar to the reduction in the comparison group (8.1 at baseline, 3.5 at 60 days). Among the pilot patients who continued an extra 30 days, mean NumDD was 2.99 at 90 days; mean NumDD in the comparison group at their post-treatment 90-day assessment was 4.04. PDA in the pilot study patients at baseline and 60 days (58.1%; 79.2%) was similar to PDA in the comparison group (61.3%; 82.1%). End-of-treatment 30-day abstention was 25.6% in pilot study patients versus 16.3% of the comparison group, not significant but favoring HealthCall-S.

### Pilot study patient perceptions of and satisfaction with HealthCall-S participation

As shown in Table 3, a substantial majority of pilot patients felt that HealthCall-S participation reminded them of their drinking goal and initial meeting with their counselor. Nearly all patients liked using HealthCall-S and felt their responses were safe, although slightly over 1/3 nevertheless had some concerns about privacy. Just over half the patients were surprised by their drinking pattern as shown in the 30-day graph. A large majority felt that HealthCall-S participation helped them remember and understand their drinking patterns, and that it increased their motivation and self-efficacy regarding drinking reduction. Of the 30 daily suggestions on cutting down drinking or maintaining abstinence (as noted in Table 1, Row 14; data available on request), 15 were rated helpful/very helpful by over two-thirds of the patients, and 13 were rated helpful/very helpful by over half the patients.

**Table 3:** **Patient feedback on HealthCall**-**S procedures at end of final appointment**

	**Always**/**Most of the time**	**Half the time**	**Infrequently**/**Never**
Reminded them of drinking goal	86.49	10.81	2.70
Reminded them of initial meeting with counselor	67.57	18.92	13.51
	**Agree**	**Not sure**	**Disagree**
Felt HealthCall-S responses were safe	94.59	5.41	0.00
Concerns about privacy using Healthcall-S	37.84	0.00	62.16
Liked using Healthcall-S	91.89	2.70	5.41
Surprised by the drinking pattern shown in 30-day graph	56.76	8.11	35.14
Helped remember drinking quantity, frequency	83.78	10.81	5.41
Helped understand drinking quantity, frequency	91.89	5.41	2.70
Increased motivation to reduce drinking	81.08	16.22	2.70
Increased confidence could reduce drinking	83.33	13.89	2.78
HealthCall graph increased interest in HealthCall-S	86.49	8.11	5.41
HealthCall graph increased perceived benefit of HealthCall-S	91.89	5.41	2.70

In the comparison group, we did not obtain quantitative data on patient reactions, but rather, conducted brief unstructured discussions with patients at the end of their 60-day meeting. While most said HealthCall was easy to use and helpful in increasing awareness of drinking and triggers, the most common suggestion was the need to introduce variety in the calls to reduce repetitiveness, information that shaped HealthCall-S development.

## Discussion

The primary aim of the pilot study was to determine the feasibility and acceptability of HealthCall for the smartphone (HealthCall-S) after a brief drinking-reduction motivational interview in alcohol dependent, minority HIV-infected patients. This study was conducted in a real-world, urban HIV primary care setting. Through 60 days of treatment, the high engagement and retention rate suggests that HealthCall-S was very acceptable to these patients. Further, the statistically significant difference between use rates in the pilot study and comparison group suggests better patient engagement in HealthCall-S than HealthCall-IVR. Further, post-intervention patient responses regarding satisfaction with HealthCall-S not only indicated that HealthCall-S participation was a positive experience, but suggested that it strengthened self-awareness, motivation and self-efficacy, the elements we wished to reinforce through HealthCall-S. Given the risk heavy drinking poses to the health and survival of HIV alcohol dependent patients and the lack of sustainable interventions for them, the pilot study offers promise for an intervention that does not require extensive personnel time and resources to administer.

In patients using HealthCall-IVR or HealthCall-S, drinking decreased from baseline to 30 days, and then decreased further between 31 and 60 days. The further decreases contrasted with drinking between 31–60 day drinking in the other arms of the randomized trial, in which drinking stayed flat (MI-only) or rebounded (attentional control). The 31- to 60-day decreases among patients using HealthCall is consistent with a randomized pilot study comparing MI + HealthCall-IVR to MI-only targeting drug use among HIV-infected patients [37]. These similarities suggest that HealthCall effects generalize across platforms and substances. Patients in MI-only arms also met with counselors but had no self-monitoring data to review. We therefore attribute the continued 31-to-60 day decline to the personalized feedback based on self-monitoring data and its discussion at 30 days, which may provide patients and counselors with a more accurate picture of the prior 30 days than patient retrospective recall, allowing for more valid planning about maintaining gains and achieving further progress.

Healthcall is theorized to work by increasing: (1) drinking awareness through self-monitoring; (2) commitment to change; and (3) self-efficacy [35]. Patients’ subjective reactions after participating in MI + HealthCall-S suggested that HealthCall-S was helpful through these mechanisms. However, to formally determine whether HealthCall effects are mediated through these constructs, they must be measured before and after HealthCall-S participation, and subjected to mediation analysis. This should be done in future studies.

At 60 days, we offered an additional 30 days of HealthCall-S participation to pilot study patients, of whom 62% continued. These patients showed lower drinking at 90 days than the comparison group, which had an assessment-only visit at 90 days. Because pilot study patients self-selected to continue, conclusions about benefits of the extra 30 days cannot be drawn. In our original proof-of-concept HealthCall pilot study [36], only 48.4% of the patients wished to continue from 60 to 90 days, leading us to select 60 days as the HealthCall “dose” for the 3-arm randomized drinking-reduction trial. For further trials, 60 days appears to remain the best duration to test, since a standardized treatment dose is needed, and a 38% treatment dropout rate by 90 days would bias results. In eventual clinical dissemination, providers and patients could adjust the HealthCall “dose”, including longer duration if the patient needed and wished to continue, “vacation” periods after stabilization of drinking reduction, and resumption if needed.

Additional study limitations are noted. First, patients were not randomly assigned to HealthCall-IVR or HealthCall-S. Potential biases that can arise from the lack of random assignment include (a) a change in the nature of patient population (demographic, clinical) treated at the clinic between the periods in which the HealthCall-IVR and HealthCall-S patients were studied; (b) a difference in the nature of the alcohol disorders in the two groups of patients; (c) a change in clinic management of the drinking outcome; or (d) a change in clinic attitudes towards MI + HealthCall after the successful outcome of the earlier randomized trial, leading to changes in the nature of the patients referred by clinic staff to the smartphone pilot study. As noted above, we carefully examined the two groups on a wide range of clinical and demographic factors. The two differences we found, shorter time since HIV diagnosis and greater severity of alcohol dependence in the pilot study group, could have led to worse outcomes in the pilot study sample. While we controlled for these two characteristics in our analyses, results for the pilot patients might have been understated due to their more serious prognostic factors. Regarding clinic management of alcohol dependence, this did not change during the two study periods. Regarding clinic attitudes, over the course of the original randomized trial, the clinic medical director and staff become increasingly positive about patient participation in our studies. However, other than the two differences we detected between comparison and pilot study patients (more recent HIV diagnosis and greater alcohol dependence severity in the pilot study patients) we know of no differences in the nature of patients referred to us across the course of that study, or differences between referrals in that study and the pilot study. We do not consider the present study to provide the same level of definitive information as a randomized trial. However, for the preliminary goals of the present pilot study, the similar or identical procedures, clinic, eligibility criteria and their assessment, outcome assessments, study personnel, and other patient characteristics suggest that the HIV alcohol dependent patients in MI + HealthCall-IVR were a reasonably informative comparison group for the HealthCall-S pilot patients.

A second limitation is that the pilot study did not include post-treatment follow-up. In the three-arm trial, follow-ups were at 90 days, 6 months, and 12 months. Exploring 12-month drinking data by HealthCall call rates showed that alcohol dependent patients with call rates at or above the median call rate drank less at 12 months (mean NumDD = 3.4, s.d. 1.4) than patients with call rates below the median (mean NumDD = 4.9, s.d. 3.3). This difference, while not significant, is consistent with better long-term drinking outcomes among HIV-infected patients with higher HealthCall engagement. A trial of HealthCall-S with post-intervention follow-up would provide information about whether the higher HealthCall-S engagement during treatment leads to significantly better long-term outcomes.

A third limitation is that much of the content (questions and statements; Table 2) for the self-monitoring component of HealthCall-S involves drinking. We do not know whether this content contributed to HealthCall-S effects, or whether daily participation in questions and statements on topics unrelated to alcohol would produce a similar drinking-reduction effect. Determining this would require a trial with a control condition involving HealthCall questions and statements without alcohol content. Such a control condition would require compensating patients to participate, and it would not provide information for the personalized feedback on drinking. While such a control condition may be of interest to theoretical researchers, we have not undertaken it because (a) the personalized drinking feedback is important, (b) payment for any HealthCall daily self-monitoring participation defeats the purpose of developing a sustainable intervention and (c) even if successful, a drinking-reduction intervention with no drinking-related content would lack face validity to patients and providers, limiting eventual dissemination.

In the future, technologies may emerge offering features even more advantageous than the smartphone. The possibility of such developments does not detract from the value of developing HealthCall-S and other smartphone-based health interventions if the interventions are based on theory that can be applied to technology as it develops. We began with the automated IVR platform for HealthCall because this technology was available. What we learned from this work enabled us to take the conceptual framework for HealthCall and apply it to the technological capacities of the smartphone to better engage patients.

A question of some interest is whether HealthCall-S is effective among HIV alcohol dependent patients after they receive a briefer drinking-reduction intervention from clinical staff without specialized training. If so, this would greatly expand the dissemination potential of HealthCall-S, since carefully supervised motivational interviewing, common in research studies but a rare intervention among medical staff in community clinics, would no longer be required.

While HealthCall-S could potentially be used to enhance brief intervention among patients in other medical settings, we have worked with HIV-infected patients because they are all ill with a disease for which the medical consequences of heavy, dependent drinking are serious. Since the total amount of time patients are involved with HealthCall is brief (20–25 minutes with a counselor at baseline, a few minutes each day for HealthCall, and 10–15 minutes with a counselor at 30 and 60 days), we have not attempted to simultaneously influence multiple HIV problems, since the possibility of successfully influencing any health behavior is uncertain if too many are targeted in the same brief intervention. However, for alcohol dependent HIV-infected patients with ART adherence problems that are clearly linked to drinking, a joint approach after minor adaptations of HealthCall-S could be successful and should be explored in a new trial.

## Conclusions

In summary, this pilot study showed significantly improved patient engagement using HealthCall-S compared to HealthCall-IVR, and considerable patient satisfaction. Results confirm that HealthCall-S is feasible, suggest that it is as effective as HealthCall-IVR in the short-term, and offer the possibility that the longer-term outcomes may be improved through greater patient engagement during treatment (although the latter clearly requires empirical testing). We suggest that the next step in better understanding the efficacy and efficiency of HealthCall-S for drinking reduction in HIV-infected patients is a larger randomized trial in conjunction with a briefer, less skilled behavioral intervention in HIV primary care clinics.

## Abbreviations

ART: Antiretroviral; MI: Motivational interviewing; IVR: Interactive voice response; HealthCall-IVR: HealthCall on the IVR platform; HealthCall-S: HealthCall on the smartphone; A-CASI: Audio computer-assisted self-interviews; AUDADIS-IV: Alcohol Use Disorders and Associated Disabilities Interview Schedule; TFLB: TimeLine FollowBacks; NumDD: Number of drinks per drinking day; PDA: Percent days abstinent; MINT: MI Network Trainer.

## Competing interests

The authors declare that they have no competing interests.

## Authors’ contributions

DH conceived of the study, participated in its design, and drafted the manuscript. EA assisted in the design, coordinated the study, and assisted with drafting the manuscript. EG performed the analyses and assisted in editing the manuscript. All authors read and approved the final manuscript.
